# Sharp estimates for the *p*-adic Hardy type operators on higher-dimensional product spaces

**DOI:** 10.1186/s13660-017-1491-z

**Published:** 2017-09-12

**Authors:** Ronghui Liu, Jiang Zhou

**Affiliations:** College of Mathematics and System Science, Xinjiang University, Urumqi, 830046 People’s Republic of China

**Keywords:** 42B20, 42B25, 42B35, Hardy type operator, sharp estimates, *p*-adic, product Lebesgue spaces

## Abstract

In this paper, we introduce the *p*-adic Hardy type operator and obtain its sharp bound on the *p*-adic Lebesgue product spaces. Meanwhile, an analogous result is computed for the *p*-adic Lebesgue product spaces with power weights. In addition, we characterize a sufficient and necessary condition which ensures that the weighted *p*-adic Hardy type operator is bounded on the *p*-adic Lebesgue product spaces. Furthermore, the *p*-adic weighted Hardy-Cesàro operator is also obtained.

## Introduction

*p*-adic numbers were introduced by Hensel at the end of the 19th century, they constitute an integral part of number theory, algebraic geometry, representation theory and other branches of modern mathematics (see [1, 2]). However, the geometry of the space $\mathbb{Q}_{p}$ is surprisingly unlike the geometry of the space $\mathbb{R}$, in particular the Archimedean axiom is not true in $\mathbb{Q}_{p}$. Therefore the field of *p*-adic numbers has natural hierarchical structures, we refer the reader to [3–5]. In recent years, theories of functions and operators from $\mathbb{Q}^{n}_{p}$ into $\mathbb{R}$ or $\mathbb{C}$ play an important role in the *p*-adic quantum mechanics, in *p*-adic analysis. Studies of the *p*-adic Hardy operators have drawn more and more attention (for example, see [6–9]).

For a prime number *p*, let $\mathbb{Q}_{p}$ be the field of *p*-adic numbers. It is defined as the completion of the field of rational numbers $\mathbb{Q}_{p}$ with respect to the non-Archimedean *p*-adic norm $\vert \cdot \vert _{p}$. This norm is defined as follows: $\vert 0 \vert _{p}=0$; if any non-zero rational number *x* is represented as $x=p^{\gamma} \frac{m}{n}$, where *γ* is an integer and the integers *m*, *n* are indivisible by *p*, then $\vert x \vert _{p}=p^{-\gamma}$. It is easy to see that the norm satisfies the following properties:

(i) $\vert x \vert _{p}\geq0$, $\forall x\in\mathbb {{Q}}_{p}$, $\vert x \vert _{p}=0\Longleftrightarrow x=0$;
(ii) $\vert xy \vert _{p}= \vert x \vert _{p} \vert y \vert _{p}$, $\forall x,y\in\mathbb{{Q}}_{p}$;
(iii) $\vert x+y \vert _{p}\leq\max\{ \vert x \vert _{p}, \vert y \vert _{p}\}$, $\forall x,y\in \mathbb{{Q}}_{p}$, and when $\vert x \vert _{p}\neq \vert y \vert _{p}$, we have $\vert x+y \vert _{p}= \max\{ \vert x \vert _{p}, \vert y \vert _{p}\}$.

It is well known that $\mathbb{Q}_{p}$ is a typical model of non-Archimedean local fields. From the standard *p*-adic analysis, we know that any non-zero element *x* of $\mathbb{Q}_{p}$ can be uniquely represented in the canonical series

$$ x=p^{\gamma}\sum_{j=0}^{\infty}a_{j}p^{j}, \quad\gamma= \gamma(x)\in\mathbb{Z}, $$

where $a_{j}$ are integrals, $0\leq a_{j}\leq p-1$, $a_{0}\neq0$. The series converges in the *p*-adic norm because $\vert a_{j}p^{j} \vert _{p}=p^{-j}$. Let $\mathbb{Z}_{p}=\{x\in\mathbb{Q}_{p}: \vert x \vert _{p}\leq1\}$ be the class of all *p*-adic integrals in $\mathbb{Q}_{p}$ and denote $\mathbb{Z} _{p}^{*}=\mathbb{Z}_{p}\backslash\{0\}$.

The space $\mathbb{Q}^{n}_{p}$ denotes a vector space over $\mathbb{Q}_{p}$ which consists of all points $x=(x_{1},x_{2},\ldots, x _{n})$, where $x_{i}\in\mathbb{Q}_{p}$, $i=1,2,\ldots,n$. The *p*-adic norm on $\mathbb{Q}^{n}_{p}$ is

$$ \vert x \vert _{p}:=\max_{1\leq i\leq n}\bigl\{ \vert x_{i} \vert _{p}\bigr\} ,\quad x\in \mathbb{Q}^{n}_{p}. $$

Denote by $B_{\gamma}(a)=\{x\in\mathbb{Q}_{p}^{n}: \vert x-a \vert _{p}\leq p ^{\gamma}\}$, the ball with center at $a\in\mathbb{Q}_{p}^{n}$ and radius $p^{\gamma}$, and by $S_{\gamma}(a)=\{x\in\mathbb{Q}_{p} ^{n}: \vert x-a \vert _{p}= p^{\gamma}\}$ the sphere with center at $a\in \mathbb{Q}_{p}^{n}$ and radius $p^{\gamma}$, $\gamma\in\mathbb{Z}$. It is clear that $S_{\gamma}(a)=B_{\gamma}(a)\setminus B_{\gamma-1}(a)$, and we set $B_{\gamma}(0)=B_{\gamma}$ and $S_{\gamma}(0)=S_{\gamma}$.

Since $\mathbb{Q}^{n}_{p}$ is a locally compact commutative group with respect to addition, it follows from the standard analysis that there exists a Harr measure *dx* on $\mathbb{Q}^{n}_{p}$, which is unique up to a positive constant factor and is translation invariant. We normalize the measure *dx* such that

$$ \int_{B_{0}(0)}dx= \bigl\vert B_{0}(0) \bigr\vert _{H}=1, $$

where $\vert B \vert _{H}$ denotes the Harr measure of a measure subset B of $\mathbb{Q}^{n}_{p}$. By simple calculation, we obtain $\vert B_{\gamma}(a) \vert =p ^{\gamma n}$, $\vert S_{\gamma}(a) \vert =p^{\gamma n}(1-p^{-n})$.

The most fundamental averaging operator is Hardy operator defined by

$$ \mathcal{H}f(x):= \frac{1}{x} \int_{0}^{x}f(t)\,dt, $$

where the function *f* is a nonnegative integrable function on $\mathbb{R}^{+}$ and $x>0$. A celebrated integral inequality, due to Hardy [10], states that

$$ \Vert \mathcal{H}f \Vert _{L^{q}(\mathbb{R}^{+})}\leq\frac {q}{q-1} \Vert f \Vert _{L ^{q}(\mathbb{R}^{+})} $$

holds for $1< q<\infty$, and the constant $\frac{q}{q-1}$ is the best.

For the multidimensional case $n\geq2 $, generally speaking, there exist two different definitions. One is the rectangle averaging operator defined by

$$ \mathfrak{H}(f) (x):= \frac{1}{x_{1}\cdots x_{m}} \int_{0}^{x_{1}} \cdots \int_{0}^{x_{m}}f(t_{1},\ldots,t_{m})\,dt_{m}\cdots dt_{1}, $$

where the function *f* is a nonnegative measurable function on $(0,\infty)^{n}$, and $x_{i}>0$, $i=1,2,\ldots,m$. The boundedness of the operator $\mathfrak{H}$ is discussed in [11–14]. $\Vert \mathfrak{H} \Vert _{L^{q}\rightarrow L^{q}}$, the norm of $\mathfrak{H}$, is $(\frac{q}{q-1})^{n}$ and obviously depends on the dimension of the space.

The other definition is the *n*-dimensional spherical averaging operator, which was introduced by Christ and Grafakos in [15] as follows:

$$ \mathcal{H}f(x):= \frac{1}{\Omega_{n} \vert x \vert ^{n}} \int _{ \vert t \vert \leq \vert x \vert }f(t)\,dt, \quad x\in\mathbb{R}^{n} \setminus\{0\}, $$

where $\Omega_{n}$ is the volume of the unit ball in $\mathbb{R}^{n}$. The norm of $\mathcal{H}$ on $L^{q}(\mathbb{R}^{n})$ was evaluated and found to be equal to that of the one-dimensional averaging operator. $\Vert \mathcal{H} \Vert _{L^{q}\rightarrow L^{q}}$, that is to say, does not depend on the dimension of the space.

In 2012, Fu *et al.* [16] defined the following *n*-dimensional *p*-adic Hardy operator:

$$ \mathcal{H}^{p}f(x):= \frac{1}{ \vert B(0, \vert x \vert _{p}) \vert _{H}} \int_{ \vert t \vert _{p}\leq \vert x \vert _{p}}f(t)\,dt, \quad x\in\mathbb{Q}^{n}_{p} \setminus \{0\}, $$

where *f* is a nonnegative measurable function on $\mathbb{Q}^{n}_{p}$, $B(0, \vert x \vert _{p})$ is a ball in $\mathbb{Q}^{n}_{p}$ with center at $0\in\mathbb{Q}^{n}_{p}$ and radius $\vert x \vert _{p}$, and they proved the sharp estimates of the *p*-adic Hardy operator on Lebesgue spaces with power weights.

In 2013, Lu *et al.* [17] gave the definition of Hardy operator on higher-dimensional product spaces as follows:

$$ \mathcal{H}_{m}(f) (x):= \Biggl(\prod_{i=1}^{m} \frac{1}{ \vert B(0, \vert x _{i} \vert ) \vert } \Biggr) \int_{ \vert y_{1} \vert < \vert x_{1} \vert }\cdots \int_{ \vert y_{m} \vert < \vert x_{m} \vert }f(y _{1},\ldots,y_{m}) \,dy_{m}\cdots{dy_{1}}, $$

where *f* is a nonnegative measurable function on $\mathbb{R}^{n_{1}} \times\mathbb{R}^{n_{2}}\times\cdots\times\mathbb{R}^{n_{m}}$, $m\in\mathbb{N}$, $n_{i}\in\mathbb{N}$, $x=(x_{1},x_{2},\ldots,x _{m})\in\mathbb{R}^{n_{1}} \times\mathbb{R}^{n_{2}}\times\cdots\times\mathbb{R}^{n_{m}}$, $x_{i}\in\mathbb{R}^{n_{i}}$ and $\prod_{i=1}^{m} \vert x_{i} \vert \neq0$. Furthermore, the corresponding operator norm on the Lebesgue product spaces with power weights was worked out.

Next, we will introduce the definition of Hardy type operator on the higher-dimensional *p*-adic product spaces as follows and discuss the boundedness and best bound on the product of *p*-adic Lebesgue spaces.

### Definition 1.1

Let $m\in\mathbb{N} $, $n_{i}\in \mathbb{N}$, $x_{i}\in\mathbb{Q}^{n_{i}}_{p}$, $i=1,\ldots,m$, and *f* be a nonnegative measurable function on $\mathbb{Q}^{n_{1}}_{p} \times \mathbb{Q}^{n_{2}}_{p}\times\cdots\times\mathbb{Q}^{n_{m}}_{p}$. The *p*-adic Hardy type operator is defined by

$$ \mathcal{H}_{m}^{p}(f) (x)= \Biggl(\prod _{i=1}^{m}\frac{1}{ \vert B(0, \vert x _{i} \vert _{p}) \vert _{H}} \Biggr) \int_{ \vert y_{1} \vert _{p}< \vert x_{1} \vert _{p}}\cdots \int_{ \vert y_{m} \vert _{p}< \vert x_{m} \vert _{p}}f(y_{1},\ldots,y_{m}) \,dy_{m}\cdots {dy_{1}}, $$

where $x=(x_{1},x_{2},\ldots,x_{m})\in\mathbb{Q}^{n_{1}}_{p} \times \mathbb{Q}^{n_{2}}_{p}\times\cdots\times\mathbb{Q}^{n_{m}}_{p}$ with $\prod_{i=1}^{m} \vert x_{i} \vert _{p}\neq0$.

In 1984, Carton-Lebrun and Fosset [18] defined the weighted Hardy operator $\mathcal{H}_{\psi}$ by

$$ \mathcal{H}_{\psi}(f) (x)= \int_{0}^{1}f(tx)\psi(t)\,dt ,\quad x\in \mathbb{R}^{n}, $$

where $\psi:[0,1]\rightarrow[0,\infty)$ is a function, and they showed the boundedness of $\mathcal{H}_{\psi}$ on Lebesgue spaces and $\operatorname{BMO}(\mathbb{R}^{n})$ spaces. Evidently the operator $\mathcal{H}_{ \psi}$ deeply depends on the nonnegative function *ψ*. For Example, when $n=1$ and $\psi(x)=1$ for $x\in[0,1]$, the operator $\mathcal{H}_{\psi}$ is just reduced to the classical Hardy operator.

In 2006, Rim and Lee [7] defined the weighted *p*-adic Hardy operator $\mathcal{H}_{\psi}^{p}$ by

$$ \mathcal{H}_{\psi}^{p}(f) (x)= \int_{\mathbb{Z}_{p}^{*}}f(tx)\psi(t)\,dt ,\quad x\in\mathbb{Q}_{p}^{n}, $$

where *ψ* is a nonnegative function defined on $\mathbb{Z}_{p} ^{*}$, and they gave the characterization of the function *ψ* for which $\mathcal{H}_{\psi}^{p}$ is bounded on $L^{q}(\mathbb{Q}_{p} ^{n})$, $1\leq q\leq\infty$, they also got the corresponding operator norm. Obviously, if $n=1$ and $\psi(x)=1$, then $\mathcal{H}_{\psi} ^{p}$ just reduces to the *p*-adic Hardy operator $\mathcal{H}^{p}$ on $\mathbb{Q}_{p}$, which is defined by

$$ \mathcal{H}^{p}f(x):=\frac{1}{ \vert x \vert _{p}} \int_{ \vert t \vert _{p}\leq \vert x \vert _{p}}f(t)\,dt, \quad x\neq0. $$

In 2013, Fu *et al.* [19] introduced the definition of weighted Hardy operator on higher-dimensional product spaces as follows:

$$ \mathcal{H}_{\varphi}^{m}(f) (x)= \int_{0}^{1}\cdots \int_{0}^{1}f(t _{1}x_{1},\ldots,t_{m}x_{m})\varphi(t_{1},\ldots,t_{m})\,dt_{1} \cdots{dt_{m}}, $$

where the nonnegative function *ψ* defined on $[0,1]\times[0,1] \times\cdots\times[0,1]$. They obtained a sufficient and necessary condition which ensures that the operator $\mathcal{H}_{\varphi}^{m}$ is bounded on the Lebesgue product spaces.

Next, we will extend the operator $\mathcal{H}_{\varphi}^{m}$ to the higher-dimensional *p*-adic product spaces.

### Definition 1.2

Let $m\in\mathbb{N} $, $n_{i}\in \mathbb{N}$, $x _{i}\in\mathbb{Q}^{n_{i}}_{p}$, $i=1,\ldots,m$, and *f* be a nonnegative measurable function on $\mathbb{Q}^{n_{1}}_{p} \times \mathbb{Q}^{n_{2}}_{p}\times\cdots\times\mathbb{Q}^{n_{m}}_{p}$. The *p*-adic weighted Hardy type operator is defined by

$$ \mathcal{H}_{\varphi,m}^{p}(f) (x)= \int_{\mathbb{Z}_{p}^{*}}\cdots \int_{\mathbb{Z}_{p}^{*}}f(t_{1}x_{1},\ldots,t_{m}x_{m})\varphi(t _{1},\ldots,t_{m})\,dt_{1}\cdots{dt_{m}}, $$

where *φ* is a nonnegative measurable function on $\mathbb{Z} ^{*}_{p} \times\mathbb{Z}^{*}_{p}\times\cdots\times\mathbb{Z}^{*} _{p}$, and $x=(x_{1},x_{2},\ldots, x_{m})\in\mathbb{Q}^{n_{1}}_{p} \times\mathbb{Q}^{n_{2}}_{p}\times\cdots\times\mathbb{Q}^{n_{m}} _{p}$.

It follows from the Fubini theorem that we can easily formulate the dual operator of $\mathcal{H}_{\varphi,m}^{p}$ and denote it by $\mathcal{H}_{\varphi,m}^{p,*}$.

### Definition 1.3

Let $m\in\mathbb{N}$, $n_{i}\in \mathbb{N}$, $x_{i}\in\mathbb{Q}^{n_{i}}_{p}$, $i=1,\ldots,m$, and *f* be a nonnegative measurable function on $\mathbb{Q}^{n_{1}}_{p} \times \mathbb{Q}^{n_{2}}_{p}\times\cdots\times\mathbb{Q}^{n_{m}}_{p}$. The dual operator of the *p*-adic weighted Hardy type operator is defined by

$$ \mathcal{H}_{\varphi,m}^{p,*}(f) (x)= \int_{\mathbb{Z}_{p}^{*}}\cdots \int_{\mathbb{Z}_{p}^{*}}\frac{f(x_{1}/ \vert t_{1} \vert _{p},\ldots,x_{m}/ \vert t _{m} \vert _{p})\varphi(t_{1},\ldots,t_{m})}{ \vert t_{1} \vert _{p}^{n}\cdots \vert t_{m} \vert _{p} ^{n}}\,dt_{1} \cdots{dt_{m}}, $$

where *φ* is a nonnegative measurable function on $\mathbb{Z} ^{*}_{p} \times\mathbb{Z}^{*}_{p}\times\cdots\times\mathbb{Z}^{*} _{p}$, and $x=(x_{1},x_{2},\ldots, x_{m})\in\mathbb{Q}^{n_{1}}_{p} \times\mathbb{Q}^{n_{2}}_{p}\times\cdots\times\mathbb{Q}^{n_{m}} _{p}$.

In 2014, Chuong and Hung[20] introduced the weighted Hardy-Cesàro operator, a more general form of $\mathcal{H}_{ \psi}$ in the real case.

Let $\psi:[0,1]\rightarrow[0,\infty)$, $s:[0,1]\rightarrow \mathbb{R}$ be measurable functions. The weighted Hardy-Cesàro operator $\mathcal{H}_{\varphi}^{s}$, associated to parameter curve $s(x,t)=s(t)x$, is defined by

$$ \mathcal{H}_{\varphi}^{s}(f)= \int_{0}^{1}f\bigl(s(t)x\bigr)\varphi(t)\,dt, $$

for all measurable functions *f* on $\mathbb{R}^{n}$.

In 2014, Hung [21] considered the form of Hardy-Cesàro operator in *p*-adic analysis

$$ \mathcal{H}_{\varphi}^{p,s}f(x):= \int_{\mathbb{Z}^{*}_{p}}f\bigl(s(t)x\bigr) \varphi(t)\,dt, $$

where $s:\mathbb{Z}^{*}_{p}\rightarrow\mathbb{Q}_{p}$ and $\psi: \mathbb{Z}^{*}_{p}\rightarrow[0,\infty)$ are measurable functions. The author investigated the boundedness of the *p*-adic analog of the weighted Hardy-Cesàro operator on weighted Lebesgue spaces and weighted BMO spaces. In each case, the corresponding operator norms are obtained. In 2016, Chuong *et al.* [22] considered the boundedness of the *p*-adic weighted Hardy-Cesàro operators and their commutators on weighted functional spaces of Morrey type, and the corresponding operator norms are also computed.

Motivated by these famous results, first we will give a higher-dimensional version of the *p*-adic weighted Hardy-Cesàro operator.

### Definition 1.4

Let $m\in\mathbb{N}$, $n_{i}\in \mathbb{N}$, $x_{i}\in\mathbb{Q}^{n_{i}}_{p}$, $i=1,\ldots,m$, and *f* be a nonnegative measurable function on $\mathbb{Q}^{n_{1}}_{p} \times \mathbb{Q}^{n_{2}}_{p}\times\cdots\times\mathbb{Q}^{n_{m}}_{p}$. The *p*-adic weighted Hardy-Cesàro type operator is defined by

$$ \mathcal{H}_{\varphi,m}^{p,s}(f) (x)= \int_{\mathbb{Z}_{p}^{*}}\cdots \int_{\mathbb{Z}_{p}^{*}}f\bigl(s(t_{1})x_{1},\ldots,s(t_{m})x_{m}\bigr)\varphi (t_{1},\ldots,t_{m})\,dt_{1}\cdots{dt_{m}}, $$

where $s:\mathbb{Z}^{*}_{p}\rightarrow\mathbb{Q}_{p}$ and $\varphi: \mathbb{Z}^{*}_{p} \times\mathbb{Z}^{*}_{p}\times\cdots\times \mathbb{Z}^{*}_{p}\rightarrow[0,\infty)$ are measurable functions, and $x=(x_{1},x_{2},\ldots,x_{m})\in\mathbb{Q}^{n_{1}}_{p} \times \mathbb{Q}^{n_{2}}_{p}\times\cdots\times\mathbb{Q}^{n_{m}}_{p}$.

The paper is organized as follows. Section 2 is devoted to the sharp estimates of $\mathcal{H}_{m}^{p}$ on the *p*-adic Lebesgue product spaces with power weights. In Section 3, we present necessary and sufficient conditions for the boundedness of the weighted Hardy type operator and its dual operator. Furthermore, the *p*-adic weighted Hardy-Cesàro type operator also has the corresponding conclusion. In Section 4, we state explicitly the main conclusions of the research.

## Sharp estimates for the *p*-adic Hardy type operator

### Theorem 2.1

*Let* $1< q<\infty$, $m\in\mathbb{N}$, $n_{i}\in \mathbb{N}$, $x_{i}\in\mathbb{Q}^{n_{i}}_{p}$, $i=1,\ldots,m$. *If* $f\in L^{q}(\mathbb{Q}^{n_{1}}_{p} \times\mathbb{Q}^{n_{2}}_{p} \times\cdots\times\mathbb{Q}^{n_{m}}_{p})$, *then the* *p*-*adic Hardy type operator* $\mathcal{H}_{m}^{p}$ *is bounded on* $L^{q}(\mathbb{Q} ^{n_{1}}_{p} \times\mathbb{Q}^{n_{2}}_{p}\times\cdots\times \mathbb{Q}^{n_{m}}_{p})$, *moreover*, *the norm of* $\mathcal{H}_{m}^{p}$ *can be obtained as follows*:

$$ \bigl\Vert \mathcal{H}_{m}^{p} \bigr\Vert _{{L^{q}(\mathbb {Q}^{n_{1}}_{p} \times \mathbb{Q}^{n_{2}}_{p}\times\cdots\times\mathbb{Q}^{n_{m}}_{p})} \rightarrow L^{q}(\mathbb{Q}^{n_{1}}_{p} \times\mathbb{Q}^{n_{2}} _{p}\times\cdots\times\mathbb{Q}^{n_{m}}_{p})}=\prod_{i=1} ^{m} \frac{1-p^{-n_{i}}}{1-p^{\frac{n_{i}}{q}-n_{i}}}. $$

We provide the following weighted extension of this result.

### Theorem 2.2

*Let* $1< q<\infty$, $m\in\mathbb{N}$, $n_{i}\in \mathbb{N}$, $x_{i}\in\mathbb{Q}^{n_{i}}_{p}$, $i=1,\ldots,m$. *If* $f\in L^{q}(\mathbb{Q}^{n_{1}}_{p} \times\mathbb{Q}^{n_{2}}_{p} \times\cdots\times\mathbb{Q}^{n_{m}}_{p}, \vert x \vert _{p}^{\vec{\alpha}})$, *where* $\vert x \vert _{p}^{\vec{\alpha}}:= \vert x \vert _{p}^{\alpha_{1}}\times \vert x \vert _{p} ^{\alpha_{2}}\times\cdots\times \vert x \vert _{p}^{\alpha_{m}}$ *and* $\alpha_{i}<(q-1)n _{i}$, *then the* *p*-*adic Hardy type operator* $\mathcal{H}_{m}^{p}$ *is bounded on* $L^{q}(\mathbb{Q}^{n_{1}}_{p} \times\mathbb{Q}^{n_{2}} _{p}\times\cdots\times\mathbb{Q}^{n_{m}}_{p}, \vert x \vert _{p}^{ \vec{\alpha}})$, *moreover*, *the norm of* $\mathcal{H}_{m}^{p}$ *can be obtained as follows*:

$$ \bigl\Vert \mathcal{H}_{m}^{p} \bigr\Vert _{{L^{q}(\mathbb {Q}^{n_{1}}_{p} \times \mathbb{Q}^{n_{2}}_{p}\times\cdots\times\mathbb{Q}^{n_{m}}_{p}, \vert x \vert _{p} ^{\vec{\alpha}})}\rightarrow L^{q}(\mathbb{Q}^{n_{1}}_{p} \times \mathbb{Q}^{n_{2}}_{p}\times\cdots\times\mathbb{Q}^{n_{m}}_{p}, \vert x \vert _{p} ^{\vec{\alpha}})}=\prod_{i=1}^{m} \frac{1-p^{-n_{i}}}{1-p^{\frac{n _{i}}{q}+\frac{\alpha_{i}}{q}-n_{i}}}. $$

When $\alpha_{i}=0$, the sharp estimate of the *p*-adic Hardy type operator will be easy to get on the *p*-adic Lebesgue product spaces, so we only provide the proof of Theorem 2.1.

### Proof

Without loss of generality, we consider only the situation when $m=2$. Actually, a similar procedure works for all $m\in\mathbb{N}$. We set

$$\begin{aligned} g_{f}(x_{1},x_{2})=\frac{1}{(1-p^{-n_{1}})} \frac{1}{(1-p^{-n_{2}})} \int_{ \vert \xi_{1} \vert _{p}=1} \int_{ \vert \xi _{2} \vert _{p}=1}f\bigl( \vert x_{1} \vert _{p}^{-1}\xi _{1}, \vert x _{2} \vert _{p}^{-1}\xi_{2}\bigr)\,d \xi_{2}\,d\xi_{1}. \end{aligned}$$

It is clear that $g_{f}(x_{1},x_{2})=g_{f}( \vert x_{1} \vert _{p}^{-1}, \vert x_{2} \vert _{p} ^{-1})$, in the following we briefly call this function a radial function on the *p*-adic Lebesgue product spaces with power weights. We have

$$\begin{aligned} \mathcal{H}_{2}^{p}(g_{f}) (x_{1},x_{2}) =& \frac{1}{ \vert B(0, \vert x_{1} \vert _{p}) \vert _{H}}\frac{1}{ \vert B(0, \vert x_{2} \vert _{p}) \vert _{H}} \int_{ \vert y_{1} \vert _{p}< \vert x_{1} \vert _{p}} \int_{ \vert y_{2} \vert _{p}< \vert x_{2} \vert _{p}}g_{f}(y _{1},y_{2}) \,dy_{2}\,dy_{1} \\ =&\frac{1}{ \vert B(0, \vert x_{1} \vert _{p}) \vert _{H}}\frac{1}{ \vert B(0, \vert x_{2} \vert _{p}) \vert _{H}} \int_{ \vert y_{1} \vert _{p}< \vert x_{1} \vert _{p}} \int_{ \vert y_{2} \vert _{p}< \vert x_{2} \vert _{p}}\frac{1}{(1-p ^{-n_{1}})}\frac{1}{(1-p^{-n_{2}})} \\ &{}\times \int_{ \vert \xi_{1} \vert _{p}=1} \int_{ \vert \xi _{2} \vert _{p}=1}f\bigl( \vert y_{1} \vert _{p} ^{-1}\xi_{1}, \vert y_{2} \vert _{p}^{-1}\xi_{2}\bigr)\,d \xi_{2}\, d\xi_{1}\,dy_{2}\,dy_{1}. \end{aligned}$$

By changing variables, $z_{i}= \vert y_{i} \vert _{p}^{-1}\xi _{i} $, $i=1,2$, we have

$$\begin{aligned} \mathcal{H}_{2}^{p}(g_{f}) (x_{1},x_{2}) =&\frac{1}{(1-p^{-n_{1}})}\frac{1}{(1-p ^{-n_{2}})} \frac{1}{ \vert B(0, \vert x_{1} \vert _{p}) \vert _{H}} \frac{1}{ \vert B(0, \vert x_{2} \vert _{p}) \vert _{H}} \\ &{}\times \int_{ \vert y_{1} \vert _{p}< \vert x_{1} \vert _{p}} \int_{ \vert y_{2} \vert _{p}< \vert x_{2} \vert _{p}} \int_{ \vert z_{1} \vert _{p}= \vert y_{1} \vert _{p}} \int_{ \vert z_{2} \vert _{p}= \vert y_{2} \vert _{p}} f(z _{1},z_{2}) \\ &{}\times \vert y_{1} \vert _{p}^{-n_{1}} \vert y_{2} \vert _{p}^{-n_{2}}\,dz_{2}\,dz_{1} \,dy_{2}\,dy _{1}. \\ =&\frac{1}{(1-p^{-n_{1}})}\frac{1}{(1-p^{-n_{2}})}\frac{1}{ \vert B(0, \vert x _{1} \vert _{p}) \vert _{H}}\frac{1}{ \vert B(0, \vert x_{2} \vert _{p}) \vert _{H}} \\ &{}\times \int_{ \vert z_{1} \vert _{p}< \vert x_{1} \vert _{p}} \int_{ \vert z_{2} \vert _{p}< \vert x_{2} \vert _{p}} \int_{ \vert y_{1} \vert _{p}= \vert z_{1} \vert _{p}} \int_{ \vert y_{2} \vert _{p}= \vert z_{2} \vert _{p}} \vert y _{1} \vert _{p}^{-n_{1}} \\ &{}\times \vert y_{2} \vert _{p}^{-n_{2}}\, dy_{2}\,dy_{1}f(z_{1},z_{2}) \,dz _{2}\,dz_{1}. \\ =&\frac{1}{ \vert B(0, \vert x_{1} \vert _{p}) \vert _{H}}\frac{1}{ \vert B(0, \vert x_{2} \vert _{p}) \vert _{H}} \int_{ \vert z_{1} \vert _{p}< \vert x_{1} \vert _{p}} \int_{ \vert z_{2} \vert _{p}< \vert x_{2} \vert _{p}}f(z_{1},z _{2}) \,dz_{2}\,dz_{1}. \\ =&\mathcal{H}_{2}^{p}(f) (x_{1},x_{2}). \end{aligned}$$

Using Hölder’s inequality, we get

$$\begin{aligned}& \Vert g \Vert _{L^{q}(\mathbb{Q}^{n_{1}}_{p} \times\mathbb {Q}^{n_{2}}_{p} , {\vec{\alpha}})} \\& \quad = \biggl( \int_{\mathbb{Q}^{n_{1}}_{p}} \int_{\mathbb{Q}^{n_{2}}_{p}} \biggl\vert \frac{1}{(1-p^{-n_{1}})}\frac{1}{(1-p ^{-n_{2}})} \\& \quad\quad {}\times \int_{ \vert \xi_{1} \vert _{p}=1} \int_{ \vert \xi_{2} \vert _{p}=1} f\bigl( \vert x_{1} \vert _{p}^{-1} \xi_{1}, \vert x_{2} \vert _{p}^{-1} \xi_{2}\bigr)\,d\xi_{2}\,d\xi_{1} \biggr\vert ^{q} \vert x _{1} \vert _{p}^{\alpha_{1}} \vert x_{2} \vert _{p}^{\alpha_{2}}\,dx_{2} \,dx_{1} \biggr)^{1/q} \\& \quad \leq \biggl( \int_{\mathbb{Q}^{n_{1}}_{p}} \int_{\mathbb {Q}^{n_{2}}_{p}}\frac{1}{(1-p ^{-n_{1}})^{q}}\frac{1}{(1-p^{-n_{2}})^{q}} \\& \quad\quad {}\times \biggl( \int_{ \vert \xi_{1} \vert _{p}=1} \int_{ \vert \xi_{2} \vert _{p}=1} \bigl\vert f\bigl( \vert x_{1} \vert _{p} ^{-1}\xi_{1}, \vert x_{2} \vert _{p}^{-1}\xi_{2}\bigr) \bigr\vert ^{q}\,d\xi_{2}\,d\xi_{1} \biggr) \\& \quad\quad {}\times \biggl( \int_{ \vert \xi_{1} \vert _{p}=1} \int_{ \vert \xi_{2} \vert _{p}=1}\,d\xi_{2}\,d\xi _{1} \biggr)^{q-1} \vert x_{1} \vert _{p}^{\alpha_{1}} \vert x_{2} \vert _{p}^{\alpha_{2}} \,dx_{2}\,dx _{1} \biggr)^{1/q} \\& \quad = \biggl( \int_{\mathbb{Q}^{n_{1}}_{p}} \int_{\mathbb{Q}^{n_{2}}_{p}}\frac{1}{(1-p ^{-n_{1}})}\frac{1}{(1-p^{-n_{2}})} \\& \quad\quad {}\times \biggl( \int_{ \vert \xi_{1} \vert _{p}=1} \int_{ \vert \xi_{2} \vert _{p}=1} \bigl\vert f\bigl( \vert x_{1} \vert _{p}^{-1}\xi_{1}, \vert x_{2} \vert _{p}^{-1}\xi_{2}\bigr) \bigr\vert ^{q}\,d\xi _{2}\,d\xi_{1} \biggr) \vert x_{1} \vert _{p}^{\alpha_{1}} \vert x_{2} \vert _{p}^{\alpha_{2}}\,dx _{2}\,dx_{1} \biggr)^{1/q} \\& \quad = \frac{1}{(1-p^{-n_{1}})^{1/q}}\frac{1}{(1-p^{-n_{2}})^{1/q}} \bigg(\int_{\mathbb {Q}^{n_{1}}_{p}}\int_{\mathbb {Q}^{n_{2}}_{p}}\bigg(\int_{|z_{1}|_{p}=|x_{1}|_{p}}\int_{|z_{2}|_{p}=|x_{2}|_{p}}\\& \quad\quad{} \times\bigl|f(z_{1},z_{2})\bigr|^{q}|x_{1}|_{p}^{-n_{1}}|x_{2}|_{p}^{-n_{2}} \,dz_{2}\,dz_{1}\bigg)|x_{1}|_{p}^{\alpha_{1}}|x_{2}|_{p}^{\alpha_{2}}\,dx_{2}\,dx_{1}\bigg)^{1/q}\\& \quad =\frac{1}{(1-p^{-n_{1}})^{1/q}}\frac{1}{(1-p^{-n_{2}})^{1/q}}\bigg(\int_{\mathbb {Q}^{n_{1}}_{p}}\int_{\mathbb {Q}^{n_{2}}_{p}}\bigg(\int_{|x_{1}|_{p}=|z_{1}|_{p}}\int_{|x_{2}|_{p}=|z_{2}|_{p}}\\& \quad\quad{} \times|x_{1}|_{p}^{\alpha_{1}-{n_{1}}}|x_{2}|_{p}^{\alpha_{2}-{n_{2}}}\,dx_{2}\,dx_{1}\bigg)|f(z_{1},z_{2})|^{q}\,dz_{2}\,dz_{1}\bigg)^{1/q}\\& \quad = \Vert f \Vert _{L^{q}(\mathbb{Q}^{n_{1}}_{p} \times\mathbb {Q}^{n_{2}}_{p} , {\vec{\alpha}})}. \end{aligned}$$

Therefore,

$$\begin{aligned} \frac{ \Vert \mathcal{H}_{2}^{p}(f) \Vert _{L^{q}(\mathbb {Q}^{n_{1}}_{p} \times \mathbb{Q}^{n_{2}}_{p} , {\vec{\alpha}})}}{ \Vert f \Vert _{L^{q}(\mathbb{Q} ^{n_{1}}_{p} \times\mathbb{Q}^{n_{2}}_{p} , {\vec{\alpha}})}}\leq \frac{ \Vert \mathcal{H}_{2}^{p}(g_{f}) \Vert _{L^{q}(\mathbb {Q}^{n_{1}}_{p} \times \mathbb{Q}^{n_{2}}_{p} , {\vec{\alpha}})}}{ \Vert g_{f} \Vert _{L^{q}( \mathbb{Q}^{n_{1}}_{p} \times\mathbb{Q}^{n_{2}}_{p} ,{\vec{\alpha}})}}. \end{aligned}$$

This implies the claim that if *f* is a radial function, then we have $g_{f}=f$. This means that the norm of the operator $\mathcal{H}_{2} ^{p}$ is equal to the norm that makes $\mathcal{H}_{2}^{p}$ restricted to the set of radial functions. Consequently, without loss of generality, it suffices to fulfil the proof of the theorem by assuming *f* is a radial function.

Substituting the variable $y_{i}= \vert x_{i} \vert _{p}^{-1}z_{i} $, $i=1,2$, we see that $\Vert \mathcal{H}_{2}^{p}(f) \Vert _{L^{q}(\mathbb {Q}^{n_{1}}_{p} \times\mathbb{Q}^{n_{2}}_{p} , {\vec{\alpha}})}$ equals

$$\begin{aligned} & \biggl( \int_{\mathbb{Q}^{n_{1}}_{p}} \int_{\mathbb{Q}^{n_{2}}_{p}} \bigl\vert \mathcal{H}_{2}^{p}(f) (x_{1},x_{2}) \bigr\vert ^{q} \vert x_{1} \vert _{p}^{\alpha_{1}} \vert x _{2} \vert _{p}^{\alpha_{2}}\,dx_{2}\,dx_{1} \biggr)^{1/q} \\ &\quad = \biggl( \int_{\mathbb{Q}^{n_{1}}_{p}} \int_{\mathbb{Q}^{n_{2}}_{p}} \biggl\vert \frac{1}{ \vert B(0, \vert x _{1} \vert _{p}) \vert _{H}}\frac{1}{ \vert B(0, \vert x_{2} \vert _{p}) \vert _{H}} \\ & \qquad {}\times \int_{ \vert y_{1} \vert _{p}< \vert x_{1} \vert _{p}} \int_{ \vert y_{2} \vert _{p}< \vert x_{2} \vert _{p}} f(y_{1},y_{2})\,dy_{2} \,dy_{1} \biggr\vert ^{q} \vert x_{1} \vert _{p}^{\alpha_{1}} \vert x_{2} \vert _{p} ^{\alpha_{2}}\,dx_{2}\,dx_{1} \biggr)^{1/q} \\ &\quad = \biggl( \int_{\mathbb{Q}^{n_{1}}_{p}} \int_{\mathbb{Q}^{n_{2}}_{p}} \biggl\vert \int_{ \vert z_{1} \vert _{p}< 1} \int_{ \vert z_{2} \vert _{p}< 1}f\bigl( \vert x_{1} \vert _{p}^{-1}z_{1}, \vert x _{2} \vert _{p}^{-1}z_{2}\bigr)\,dz_{2} \,dz_{1} \biggr\vert ^{q} \vert x_{1} \vert _{p}^{\alpha_{1}} \vert x_{2} \vert _{p} ^{\alpha_{2}}\,dx_{2}\,dx_{1} \biggr)^{1/q}. \end{aligned}$$

Using the generalized Minkowski’s inequality and noting that *f* is a radial function, we have $\Vert \mathcal{H}_{2}^{p}(f) \Vert _{L^{q}( \mathbb{Q}^{n_{1}}_{p} \times\mathbb{Q}^{n_{2}}_{p} , {\vec{\alpha}})}$ that is not greater than

$$\begin{aligned}& \biggl( \int_{\mathbb{Q}^{n_{1}}_{p}} \int_{\mathbb{Q}^{n_{2}}_{p}} \biggl\vert \int_{ \vert z_{1} \vert _{p}< 1} \int_{ \vert z_{2} \vert _{p}< 1}f\bigl( \vert z_{1} \vert _{p}^{-1}x_{1}, \vert z _{2} \vert _{p}^{-1}x_{2}\bigr) \biggr\vert \,dz_{2}\,dz_{1} \vert ^{q} \vert x_{1} \vert _{p}^{\alpha_{1}} \vert x_{2} \vert _{p} ^{\alpha_{2}}\,dx_{2}\,dx_{1} \biggr)^{1/q} \\& \quad \leq \int_{ \vert z_{1} \vert _{p}< 1} \int_{ \vert z_{2} \vert _{p}< 1} \biggl( \int_{\mathbb{Q}^{n_{1}}_{p}} \int_{\mathbb{Q}^{n_{2}}_{p}} \bigl\vert f\bigl( \vert z_{1} \vert _{p} ^{-1}x_{1}, \vert z_{2} \vert _{p}^{-1}x_{2}\bigr) \bigr\vert ^{q} \vert x_{1} \vert _{p}^{\alpha_{1}} \vert x_{2} \vert _{p} ^{\alpha_{2}} \,dx_{2}\,dx_{1} \biggr)^{1/q}\,dz_{2} \,dz_{1} \\& \quad = \int_{ \vert z_{1} \vert _{p}< 1} \int_{ \vert z_{2} \vert _{p}< 1} \biggl( \int_{\mathbb{Q}^{n_{1}}_{p}} \int_{\mathbb{Q}^{n_{2}}_{p}} \bigl\vert f(y_{1},y _{2}) \bigr\vert ^{q}\frac{ \vert y_{1} \vert _{p}^{\alpha _{1}}}{ \vert z_{1} \vert _{p}^{\alpha_{1}}}\frac{ \vert y _{2} \vert _{p}^{\alpha_{2}}}{ \vert z_{2} \vert _{p}^{\alpha_{2}}} \,dy_{2}\,dy_{1} \biggr)^{1/q} \vert z _{1} \vert _{p}^{-n_{1}/q} \vert z_{2} \vert _{p}^{-n_{2}/q}\,dz_{2}\,dz_{1} \\& \quad = \int_{ \vert z_{1} \vert _{p}< 1} \int_{ \vert z_{2} \vert _{p}< 1} \vert z_{1} \vert _{p}^{\frac{-{{n _{1}}-{\alpha_{1}}}}{q}} \vert z_{2} \vert _{p}^{ \frac{-{{n_{2}}-{\alpha_{2}}}}{q}}\,dz_{2}\,dz_{1} \Vert f \Vert _{L^{q}(\mathbb{Q} ^{n_{1}}_{p} \times\mathbb{Q}^{n_{2}}_{p} , {\vec{\alpha}})} \\& \quad =\prod_{i=1}^{2}\frac{1-p^{-n_{i}}}{1-p^{\frac{n_{i}}{q}+\frac{ \alpha_{i}}{q}-n_{i}}} \Vert f \Vert _{L^{q}(\mathbb {Q}^{n_{1}}_{p} \times \mathbb{Q}^{n_{2}}_{p} , {\vec{\alpha}})}. \end{aligned}$$

Therefore,

$$\begin{aligned} \bigl\Vert \mathcal{H}_{2}^{p} \bigr\Vert _{{L^{q}(\mathbb {Q}^{n_{1}}_{p} \times \mathbb{Q}^{n_{2}}_{p}, \vert x \vert _{p}^{\vec{\alpha }})}\rightarrow L^{q}( \mathbb{Q}^{n_{1}}_{p} \times\mathbb{Q}^{n_{2}}_{p}, \vert x \vert _{p}^{ \vec{\alpha}})}\leq\prod_{i=1}^{2} \frac{1-p^{-n_{i}}}{1-p ^{\frac{n_{i}}{q}+\frac{\alpha_{i}}{q}-n_{i}}}. \end{aligned}$$

Now let us prove that our estimate is sharp. For $0<\varepsilon<\min \{1,{(q-1)n_{1}}/q,{(q-1)n_{2}}/q \}$, we take

$$ f_{\varepsilon}(x_{1},x_{2})= \vert x_{1} \vert _{p}^{\frac {-{{n_{1}}-{\alpha _{1}}}}{q}+\varepsilon} \vert x_{2} \vert _{p}^{ \frac{-{{n_{2}}-{\alpha_{2}}}}{q}+\varepsilon}\chi_{\{ \vert x_{1} \vert _{p}< 1 \}}\chi_{\{ \vert x_{2} \vert _{p}< 1\}}(x_{1},x_{2}). $$

It follows from the elementary calculation that $\Vert f_{\varepsilon} \Vert _{L^{q}(\mathbb{Q}^{n_{1}}_{p} \times\mathbb{Q}^{n_{2}}_{p} , {\vec{\alpha}})}$ is

$$\begin{aligned} & \biggl( \int_{ \vert x_{1} \vert _{p}< 1} \int_{ \vert x_{2} \vert _{p}< 1} \vert x_{1} \vert _{p}^{(\varepsilon -\frac{{n_{1}}+{\alpha_{1}}}{q})q} \vert x_{2} \vert _{p}^{(\varepsilon-\frac{ {n_{2}}+{\alpha_{2}}}{q})q} \vert x \vert _{p}^{\vec{\alpha }} \,dx_{2}\,dx_{1} \biggr)^{1/q} \\ &\quad = \biggl( \int_{ \vert x_{1} \vert _{p}< 1} \vert x_{1} \vert _{p}^{(\varepsilon- \frac{{n_{1}}}{q})q} \biggr)^{1/q} \biggl( \int_{ \vert x_{2} \vert _{p}< 1} \vert x_{2} \vert _{p} ^{(\varepsilon-\frac{{n_{2}}}{q})q} \biggr)^{1/q} \\ &\quad = \biggl(\frac{1-p^{-n_{1}}}{1-p^{-q\varepsilon}} \biggr)^{1/q} \biggl(\frac{1-p ^{-n_{2}}}{1-p^{-q\varepsilon}} \biggr)^{1/q}. \end{aligned}$$

We rewrite $\mathcal{H}_{2}^{p}(f_{\varepsilon})$

$$\begin{aligned} &\mathcal{H}_{2}^{p}(f_{\varepsilon}) (x_{1},x_{2}) \\ &\quad =\frac{1}{ \vert B(0, \vert x _{1} \vert _{p}) \vert _{H}}\frac{1}{ \vert B(0, \vert x_{2} \vert _{p}) \vert _{H}} \int_{ \vert y_{1} \vert _{p}< \vert x_{1} \vert _{p}} \int_{ \vert y_{2} \vert _{p}< \vert x_{2} \vert _{p}}f(y_{1},y _{2}) \,dy_{2}\,dy_{1} \\ &\quad =\frac{1}{ \vert B(0, \vert x_{1} \vert _{p}) \vert _{H}}\frac{1}{ \vert B(0, \vert x_{2} \vert _{p}) \vert _{H}} \int_{ \vert z_{1} \vert _{p}< 1} \int_{ \vert z_{2} \vert _{p}< 1}f\bigl(z_{1} \vert x_{1} \vert _{p}^{-n_{1}},z _{2} \vert x_{2} \vert _{p}^{-n_{2}}\bigr)\,dz_{2}\,dz_{1} \\ &\quad =\frac{ \vert x_{1} \vert _{p}^{\frac{-{{n_{1}}-{\alpha _{1}}}}{q}+\varepsilon}}{ \vert B(0, \vert x _{1} \vert _{p}) \vert _{H}}\frac{ \vert x_{1} \vert _{p}^{\frac{-{{n_{1}}-{\alpha_{1}}}}{q}+ \varepsilon}}{ \vert B(0, \vert x_{2} \vert _{p}) \vert _{H}} \int_{ \vert z_{1} \vert _{p}< 1} \int_{ \vert z_{2} \vert _{p}< 1} \vert z_{1} \vert _{p}^{\frac{-{{n_{1}}-{\alpha_{1}}}}{q}+ \varepsilon} \vert z_{2} \vert _{p}^{\frac{-{{n_{2}}-{\alpha _{2}}}}{q}+\varepsilon }\,dz_{2}\,dz_{1}. \end{aligned}$$

Thus, we estimate the norm of $\Vert \mathcal {H}_{2}^{p}(f_{\varepsilon}) \Vert _{L^{q}(\mathbb{Q}^{n_{1}}_{p} \times\mathbb {Q}^{n_{2}}_{p} , {\vec{\alpha}})}$ as follows:

$$\begin{aligned}& \bigl\Vert \mathcal{H}_{2}^{p}(f_{\varepsilon}) \bigr\Vert _{L^{q}(\mathbb{Q}^{n_{1}} _{p} \times\mathbb{Q}^{n_{2}}_{p} , {\vec{\alpha}})}^{q} \\& \quad = \int_{\mathbb{Q}^{n_{1}}_{p}} \int_{\mathbb{Q}^{n_{2}}_{p}} \biggl\vert \int_{\{ \vert z_{1} \vert _{p}< 1, \vert z_{1} \vert _{p}< \frac{1}{ \vert x_{1} \vert _{p}}\}} \int_{\{ \vert z_{2} \vert _{p}< 1, \vert z_{2} \vert _{p}< \frac{1}{ \vert x_{2} \vert _{p}}\}} \vert z_{1} \vert _{p} ^{\frac{-{{n_{1}}-{\alpha_{1}}}}{q}+\varepsilon} \\& \quad\quad {}\times \vert z_{2} \vert _{p}^{\frac{-{{n_{2}}-{\alpha _{2}}}}{q}+\varepsilon}\,dz _{2}\,dz_{1} \biggr\vert ^{q} \vert x_{1} \vert _{p}^{q\varepsilon-n_{1}-\alpha_{1}} \vert x_{2} \vert _{p} ^{q\varepsilon-n_{2}-\alpha_{2}} \vert x \vert _{p}^{\vec {\alpha}} \,dx_{2}\,dx_{1} \\& \quad \geq \int_{ \vert x_{1} \vert _{p}< 1} \int_{ \vert x_{2} \vert _{p}< 1} \biggl\vert \int_{ \vert z_{1} \vert _{p}< 1} \int_{ \vert z_{2} \vert _{p}< 1} \vert z_{1} \vert _{p}^{\frac{-{{n_{1}}-{\alpha_{1}}}}{q}+ \varepsilon} \\& \quad\quad {}\times \vert z_{2} \vert _{p}^{\frac{-{{n_{2}}-{\alpha _{2}}}}{q}+\varepsilon}\,dz _{2}\,dz_{1} \biggr\vert ^{q} \vert x_{1} \vert _{p}^{q\varepsilon-n_{1}} \vert x_{2} \vert _{p}^{q\varepsilon -n_{2}} \vert x \vert _{p}^{\vec{\alpha}} \,dx_{2}\,dx_{1} \\& \quad = \frac{1-p^{-n_{1}}}{1-p^{-q\varepsilon}}\frac{1-p^{-n_{2}}}{1-p ^{-q\varepsilon}} \biggl( \int_{ \vert z_{1} \vert _{p}< 1} \int _{ \vert z_{2} \vert _{p}< 1} \vert z_{1} \vert _{p} ^{\frac{-{{n_{1}}-{\alpha_{1}}}}{q}+\varepsilon} \vert z_{2} \vert _{p}^{\frac{- {{n_{2}}-{\alpha_{2}}}}{q}+\varepsilon} \,dz_{2}\,dz_{1} \biggr)^{q} \\& \quad = \Biggl(\prod_{i=1}^{m} \frac{1-p^{-n_{i}}}{1-p^{ \frac{n_{i}}{q}+\frac{\alpha_{i}}{q}-n_{i}-\varepsilon}} \Biggr)^{q} \Vert f \Vert _{L^{q}(\mathbb{Q}^{n_{1}}_{p} \times\mathbb {Q}^{n_{2}}_{p} , {\vec{\alpha}})}^{q}. \end{aligned}$$

Therefore,

$$\begin{aligned} \bigl\Vert \mathcal{H}_{2}^{p} \bigr\Vert _{{L^{q}(\mathbb {Q}^{n_{1}}_{p} \times \mathbb{Q}^{n_{2}}_{p}, \vert x \vert _{p}^{\vec{\alpha }})}\rightarrow L^{q}( \mathbb{Q}^{n_{1}}_{p} \times\mathbb{Q}^{n_{2}}_{p}, \vert x \vert _{p}^{ \vec{\alpha}})}\geq\prod_{i=1}^{2} \frac{1-p^{-n_{i}}}{1-p ^{\frac{n_{i}}{q}+\frac{\alpha_{i}}{q}-n_{i}-\varepsilon}}. \end{aligned}$$

Consequently, using the definition of the norm of the operator and letting $\varepsilon\rightarrow0$, we conclude that

$$\begin{aligned} \bigl\Vert \mathcal{H}_{2}^{p} \bigr\Vert _{{L^{q}(\mathbb {Q}^{n_{1}}_{p} \times \mathbb{Q}^{n_{2}}_{p}, \vert x \vert _{p}^{\vec{\alpha }})}\rightarrow L^{q}( \mathbb{Q}^{n_{1}}_{p} \times\mathbb{Q}^{n_{2}}_{p}, \vert x \vert _{p}^{ \vec{\alpha}})}\geq\prod_{i=1}^{2} \frac{1-p^{-n_{i}}}{1-p ^{\frac{n_{i}}{q}+\frac{\alpha_{i}}{q}-n_{i}}}. \end{aligned}$$

This finishes the proof of the theorem. □

## Necessary and sufficient conditions for the boundedness of the weighted Hardy type operator

### Theorem 3.1

*Let* $1\leq q\leq\infty$, $m\in\mathbb{N}$, $n_{i}\in\mathbb{N}$, $x_{i}\in\mathbb{Q}^{n_{i}}_{p}$, $i=1,\ldots,m$. *φ* *is a nonnegative measurable function on* $\mathbb{Z} ^{*}_{p} \times\mathbb{Z}^{*}_{p}\times\cdots\times\mathbb{Z}^{*} _{p}$. *If* $f\in L^{q}(\mathbb{Q}^{n_{1}}_{p} \times\mathbb{Q}^{n_{2}} _{p}\times\cdots\times\mathbb{Q}^{n_{m}}_{p})$, *then the* *p*-*adic weighted Hardy type operator* $\mathcal{H}_{\varphi,m}^{p}$ *is bounded on* $L^{q}(\mathbb{Q}^{n_{1}}_{p} \times\mathbb{Q}^{n_{2}}_{p}\times\cdots\times\mathbb{Q}^{n_{m}}_{p})$ *if and only if*

$$ \int_{\mathbb{Z}_{p}^{*}}\cdots \int_{\mathbb{Z}_{p}^{*}} \vert t_{1} \vert _{p} ^{-n_{1}/q}\cdots \vert t_{m} \vert _{p}^{-n_{m}/q} \varphi (t_{1},\ldots,t_{m})\,dt _{1} \cdots{dt_{m}}< \infty. $$

### Proof

Since the case $q=\infty$ is trivial, it suffices to consider the case $1\leq q<\infty$.

We first prove the sufficiency of Theorem 3.1. Assume

$$ \int_{\mathbb{Z}_{p}^{*}}\cdots \int_{\mathbb{Z}_{p}^{*}} \vert t_{1} \vert _{p} ^{-n_{1}/q}\cdots \vert t_{m} \vert _{p}^{-n_{m}/q} \varphi (t_{1},\ldots,t_{m})\,dt _{1} \cdots{dt_{m}}< \infty. $$

By the generalized Minkowski’s inequality, we get $\Vert \mathcal{H}_{ \varphi,m}^{p}(f) \Vert _{L^{q}(\mathbb{Q}_{p}^{n_{1}} \times \mathbb{Q} _{p}^{n_{2}}\times\cdots\times\mathbb{Q}_{p}^{n_{m}} )}$ that is not greater than

$$\begin{aligned} \int_{\mathbb{Z}_{p}^{*}}\cdots \int_{\mathbb{Z}_{p}^{*}} \biggl( \int_{\mathbb{Q}^{n_{1}}_{p} }\cdots \int_{\mathbb{Q}^{n_{m}}_{p}} \bigl\vert f(t _{1}x_{1},\ldots,t_{m}x_{m}) \bigr\vert ^{q} \,dx_{m}\cdots dx_{1} \biggr)^{1/q} \varphi(t_{1},\ldots,t_{m})\,dt_{1} \cdots{dt_{m}}. \end{aligned}$$

Making the change of variables $y_{i}=x_{i}t_{i}$, $i=1,\ldots,m$, we have

$$\begin{aligned} & \int_{\mathbb{Z}_{p}^{*}}\cdots \int_{\mathbb{Z}_{p}^{*}} \biggl( \int_{\mathbb{Q}^{n_{1}}_{p} }\cdots \int_{\mathbb{Q}^{n_{m}}_{p}} \bigl\vert f(y _{1},\ldots,y_{m}) \bigr\vert ^{q} \,dy_{m} \cdots dy_{1} \biggr)^{1/q} \\ &\quad\quad{}\times \vert t_{1} \vert _{p} ^{-n_{1}/q}\cdots \vert t_{m} \vert _{p}^{-n_{m}/q}\varphi (t_{1},\ldots,t_{m}) \,dt _{1}\cdots{dt_{m}} \\ &\quad \leq \Vert f \Vert _{L^{q}(\mathbb{Q}_{p}^{n_{1}} \times \mathbb{Q}_{p}^{n _{2}}\times\cdots\times\mathbb{Q}_{p}^{n_{m}} )} \int_{\mathbb{Z}_{p}^{*}} \cdots \int_{\mathbb{Z}_{p}^{*}} \vert t_{1} \vert _{p}^{-n_{1}/q}\cdots \vert t_{m} \vert _{p} ^{-n_{m}/q}\varphi(t_{1},\ldots,t_{m}) \,dt_{1}\cdots{dt_{m}}. \end{aligned}$$

Since the inequality

$$ \int_{\mathbb{Z}_{p}^{*}}\cdots \int_{\mathbb{Z}_{p}^{*}} \vert t_{1} \vert _{p} ^{-n_{1}/q}\cdots \vert t_{m} \vert _{p}^{-n_{m}/q} \varphi (t_{1},\ldots,t_{m})\,dt _{1} \cdots{dt_{m}}< \infty $$

holds, this immediately implies that the operator $\mathcal{H}_{ \varphi,m}^{p} $ is bounded on $L^{q}(\mathbb{Q}_{p}^{n_{1}} \times \mathbb{Q}_{p}^{n_{2}}\times\cdots\times\mathbb{Q}_{p}^{n_{m}})$, and

$$ \begin{gathered} \bigl\Vert \mathcal{H}_{\varphi,m}^{p} \bigr\Vert _{L^{q}(\mathbb {Q}_{p}^{n_{1}} \times \mathbb{Q}_{p}^{n_{2}}\times\cdots\times\mathbb{Q}_{p}^{n_{m}} )\rightarrow L^{q}(\mathbb{Q}_{p}^{n_{1}} \times\mathbb{Q}_{p}^{n_{2}}\cdots \times\mathbb{Q}_{p}^{n_{m}} )} \\ \quad \leq \int_{\mathbb{Z}_{p}^{*}}\cdots \int_{\mathbb{Z}_{p}^{*}} \vert t_{1} \vert _{p}^{-n_{1}/q}\cdots \vert t_{m} \vert _{p}^{-n _{m}/q}\varphi(t_{1},\ldots,t_{m}) \,dt_{1}\cdots{dt_{m}}. \end{gathered} $$

This completes the proof of the sufficiency of Theorem 3.1.

Next we prove the necessary of Theorem 3.1, if $\mathcal{H}_{\varphi ,m}^{p} $ is bounded on $L^{q}(\mathbb{Q}_{p}^{n_{1}} \times \mathbb{Q}_{p}^{n_{2}}\times\cdots\times\mathbb{Q}_{p}^{n_{m}})$, then there exists a constant $C>0$ such that

$$ \bigl\Vert \mathcal{H}_{\varphi,m}^{p}(f) \bigr\Vert _{L^{q}(\mathbb {Q}_{p}^{n_{1}} \times\mathbb{Q}_{p}^{n_{2}}\times\cdots\times\mathbb{Q}_{p}^{n_{m}} )}\leq C \Vert f \Vert _{L^{q}(\mathbb{Q}_{p}^{n_{1}} \times\mathbb {Q}_{p}^{n_{2}} \times\cdots\times\mathbb{Q}_{p}^{n_{m}} )}. $$

Now, for any $0<\varepsilon<1$ and $\vert \varepsilon \vert _{p}>1$, we take

$$\begin{aligned} f_{i}^{\varepsilon}(x)= \textstyle\begin{cases} 0, & \vert x_{i} \vert _{p}< 1 , \\ \vert x_{i} \vert _{p}^{-\frac{n_{i}}{q}-\varepsilon}, & \vert x_{i} \vert _{p}\geq1 , \end{cases}\displaystyle \end{aligned}$$

and

$$ f_{\varepsilon}(x_{1},x_{2},\ldots,x_{m})= \prod_{i=1}^{m}f _{i}^{\varepsilon}(x). $$

Then a straightforward computation leads to

$$ \Vert f_{\varepsilon} \Vert _{L^{q}(\mathbb{Q}_{p}^{n_{1}} \times\mathbb{Q} _{p}^{n_{2}}\times\cdots\times\mathbb{Q}_{p}^{n_{m}} )}^{q}=\prod _{i=1}^{m}\frac{1-p^{-n_{i}}}{1-p^{-\varepsilon q}}. $$

Obviously, $\mathcal{H}_{\varphi,m}^{p}(f)(x_{1},x_{2},\ldots,x_{m})=0 $ while $\vert x_{i} \vert _{p}<1$. Moreover, we also see that, if ${\vert x_{i} \vert _{p}\geq1}$, $\mathcal{H}_{\varphi ,m}^{p}(f)(x_{1},x_{2},\ldots,x_{m})$ equals

$$ \begin{gathered} \vert x_{1} \vert _{p}^{-\frac{n_{1}}{q}-\varepsilon}\cdots \vert x_{m} \vert _{p}^{-\frac{n _{m}}{q}-\varepsilon} \int_{\frac{1}{ \vert x_{1} \vert _{p}}\leq \vert t_{1} \vert _{p}\leq1} \cdots \int_{\frac{1}{ \vert x_{m} \vert _{p}}\leq \vert t_{m} \vert _{p}\leq1} \vert t_{1} \vert _{p} ^{-\frac{n_{1}}{q}-\varepsilon}\cdots \vert t_{m} \vert _{p}^{-\frac{n_{m}}{q}- \varepsilon} \\ \quad{}\times \varphi(t_{1},\ldots,t_{m})\,dt_{m} \cdots{dt_{1}}. \end{gathered} $$

Since the inequality

$$ \bigl\Vert \mathcal{H}_{\varphi,m}^{p}(f) \bigr\Vert _{L^{q}(\mathbb {Q}_{p}^{n_{1}} \times\mathbb{Q}_{p}^{n_{2}}\times\cdots\times\mathbb{Q}_{p}^{n_{m}} )}\leq C \Vert f \Vert _{L^{q}(\mathbb{Q}_{p}^{n_{1}} \times\mathbb {Q}_{p}^{n_{2}} \times\cdots\times\mathbb{Q}_{p}^{n_{m}} )} $$

applies to $f_{\varepsilon}$, we have

$$\begin{aligned}& C^{p} \Vert f \Vert ^{q}_{L^{q}(\mathbb{Q}_{p}^{n_{1}} \times\mathbb{Q}_{p} ^{n_{2}}\times\cdots\times\mathbb{Q}_{p}^{n_{m}} )} \\& \quad \geq \bigl\Vert \mathcal{H}_{ \varphi,m}^{p} \bigr\Vert ^{q}_{L^{q}(\mathbb{Q}_{p}^{n_{1}} \times \mathbb{Q} _{p}^{n_{2}}\times\cdots\times\mathbb{Q}_{p}^{n_{m}} )} \\& \quad \geq \int_{ \vert x_{1} \vert _{p}\geq1}\cdots \int_{ \vert x_{m} \vert _{p}\geq1} \bigl( \vert x _{1} \vert _{p}^{-\frac{n_{1}}{q}-\varepsilon}\cdots \vert x_{m} \vert _{p}^{-\frac{n _{m}}{q}-\varepsilon} \bigr)^{q} \\& \quad\quad{} \times \biggl( \int_{\frac{1}{ \vert x_{1} \vert _{p}}\leq \vert t_{1} \vert _{p}\leq1} \cdots \int_{\frac{1}{ \vert x_{m} \vert _{p}}\leq \vert t_{m} \vert _{p}\leq1} \vert t_{1} \vert _{p} ^{-\frac{n_{1}}{q}-\varepsilon}\cdots \vert t_{m} \vert _{p}^{-\frac{n_{m}}{q}- \varepsilon} \\& \quad\quad{}\times \varphi(t_{1},\ldots,t_{m})\,dt_{m} \cdots{dt_{1}} \biggr)^{q}\,dx _{m} \cdots{dx_{1}} \\& \quad \geq \int_{ \vert x_{1} \vert _{p}\geq \vert \varepsilon \vert _{p}}\cdots \int_{ \vert x_{m} \vert _{p}\geq \vert \varepsilon \vert _{p}} \bigl( \vert x_{1} \vert _{p}^{-{n_{1}}- \varepsilon{q}}\cdots \vert x_{m} \vert _{p}^{-{n_{m}}-\varepsilon{q}} \bigr) \\& \quad\quad{} \times \biggl( \int_{\frac{1}{ \vert \varepsilon \vert _{p}}\leq \vert t_{1} \vert _{p}\leq1} \cdots \int_{\frac{1}{ \vert \varepsilon \vert _{p}}\leq \vert t_{m} \vert _{p}\leq1} \vert t _{1} \vert _{p}^{-\frac{n_{1}}{q}-\varepsilon}\cdots \vert t_{m} \vert _{p}^{-\frac{n _{m}}{q}-\varepsilon} \\& \quad\quad{}\times \varphi(t_{1},\ldots,t_{m}) \,dt_{m}\cdots{dt _{1}} \biggr)^{q} \,dx_{m}\cdots{dx_{1}} \\& \quad = \Vert f_{\varepsilon} \Vert _{L^{q}(\mathbb {Q}_{p}^{n_{1}} \times \mathbb{Q}_{p}^{n_{2}}\times\cdots\times\mathbb{Q}_{p}^{n_{m}} )}^{q} \vert \varepsilon \vert _{p}^{-\varepsilon mq} \\& \quad\quad{} \times \biggl( \int_{\frac{1}{ \vert \varepsilon \vert _{p}}\leq \vert t_{1} \vert _{p}\leq1} \cdots \int_{\frac{1}{ \vert \varepsilon \vert _{p}}\leq \vert t_{m} \vert _{p}\leq1} \vert t _{1} \vert _{p}^{-\frac{n_{1}}{q}-\varepsilon}\cdots \vert t_{m} \vert _{p}^{-\frac{n _{m}}{q}-\varepsilon}\varphi(t_{1},\ldots,t_{m}) \,dt_{m}\cdots{dt _{1}} \biggr)^{q}. \end{aligned}$$

Therefore,

$$\begin{aligned} & \int_{\frac{1}{ \vert \varepsilon \vert _{p}}\leq \vert t_{1} \vert _{p}\leq1}\cdots \int_{\frac{1}{ \vert \varepsilon \vert _{p}}\leq \vert t_{m} \vert _{p}\leq1} \vert t_{1} \vert _{p} ^{-\frac{n_{1}}{q}-\varepsilon}\cdots \vert t_{m} \vert _{p}^{-\frac{n_{m}}{q}- \varepsilon} \varphi(t_{1},\ldots,t_{m})\,dt_{m} \cdots{dt_{1}}\leq \frac{C}{ \vert {\varepsilon \vert _{p}}^{\varepsilon m}}. \end{aligned}$$

Now take $\varepsilon=p^{-k}$, $k=1,2,\ldots$ . Then $\vert \varepsilon \vert _{p}=p ^{k}>1$. Letting *k* approach ∞, then *ε* approaches 0 and $\vert \varepsilon \vert _{p}^{\varepsilon m}=p^{\frac {km}{p^{k}}}$ approaches 1. Then by Fatou’s lemma, we obtain

$$ \int_{\mathbb{Z}_{p}^{*}}\cdots \int_{\mathbb{Z}_{p}^{*}} \vert t_{1} \vert _{p} ^{-n_{1}/q}\cdots \vert t_{m} \vert _{p}^{-n_{m}/q} \varphi (t_{1},\ldots,t_{m})\,dt _{1} \cdots{dt_{m}}< \infty. $$

Thus, the proof of Theorem 3.1 is completed. □

Since the operator $\mathcal{H}_{\varphi,m}^{p,*}$ is the dual operator of $\mathcal{H}_{\varphi,m}^{p}$, we can immediately deduce the following result.

### Theorem 3.2

*Let* $1\leq q<\infty$, $m\in\mathbb{N}$, $n_{i}\in\mathbb{N}$, *and* *φ* *be a nonnegative measurable function on* $\mathbb{Z}^{*}_{p} \times\mathbb{Z}^{*}_{p}\times\cdots\times\mathbb{Z}^{*}_{p}$, $i=1,\ldots,m$. *If* $f\in L^{q}( \mathbb{Q}^{n_{1}}_{p} \times\mathbb{Q}^{n_{2}}_{p}\times\cdots\times\mathbb{Q}^{n_{m}}_{p})$, *then the* *p*-*adic weighted Hardy type operator* $\mathcal{H}_{\varphi,m}^{p,*}$ *is bounded on* $L^{q}( \mathbb{Q}^{n_{1}}_{p} \times\mathbb{Q}^{n_{2}}_{p}\times\cdots\times\mathbb{Q}^{n_{m}}_{p})$ *if and only if*

$$ \int_{\mathbb{Z}_{p}^{*}}\cdots \int_{\mathbb{Z}_{p}^{*}} \vert t_{1} \vert _{p} ^{-\frac{(q-1)n_{1}}{q}}\cdots \vert t_{m} \vert _{p}^{-\frac {(q-1)n_{m}}{q}} \varphi(t_{1},\ldots,t_{m})\,dt_{1} \cdots{dt_{m}}< \infty. $$

### Remark 3.1

Using methods similar to Theorem 3.1, we can easily see the proof of Theorem 3.2. So we omit the details of the proof. In particular, if $\varphi=1$, we know that the operator $\mathcal{H} _{\varphi,m}^{p}$ is not bounded on $L^{1}(\mathbb{Q}^{n_{1}}_{p} \times\mathbb{Q}^{n_{2}}_{p}\times\cdots\times\mathbb{Q}^{n_{m}} _{p})$. In the same way, when $\varphi=1$, we can also deduce that $\mathcal{H}_{\varphi,m}^{p,*}$ is not bounded on $L^{\infty}( \mathbb{Q}^{n_{1}}_{p} \times\mathbb{Q}^{n_{2}}_{p}\times\cdots\times\mathbb{Q}^{n_{m}}_{p})$.

We note that in [23], the authors proved the sharp bounds of a weighted multilinear Hardy-Cesàro operator on the product of Lebesgue spaces and central Morrey spaces. They also proved sufficient and necessary conditions of the weight functions so that the commutators of weighted multilinear Hardy-Cesàro operator with symbols in central BMO spaces.

Inspired by the paper, we will consider sufficient and necessary conditions of the weighted Hardy-Cesàro type operator $\mathcal{H}_{\varphi,m}^{p,s}$ on the *p*-adic Lebesgue product spaces.

### Theorem 3.3

*Suppose* $1\leq q<\infty$, $m\in\mathbb{N} $, $n_{i} \in\mathbb{N}$. *Let* $s:\mathbb{Z}^{*}_{p}\rightarrow\mathbb{Q}_{p}$ *be a measurable function such that* $\vert s(t_{i}) \vert _{p}\geq \vert t_{i} \vert _{p} ^{\beta}$ *for some real* *β* *and almost everywhere* $t_{i}\in \mathbb{Z}^{*}_{p}$. *If* $f\in L^{q}(\mathbb{Q}^{n_{1}}_{p} \times \mathbb{Q}^{n_{2}}_{p}\times\cdots\times\mathbb{Q}^{n_{m}}_{p})$, *then the* *p*-*adic weighted Hardy*-*Cesàro type operator* $\mathcal{H}_{\varphi,m}^{p,s}$ *is bounded on* $L^{q}(\mathbb{Q}^{n _{1}}_{p} \times\mathbb{Q}^{n_{2}}_{p}\times\cdots\times \mathbb{Q}^{n_{m}}_{p})$ *if and only if*

$$ \int_{\mathbb{Z}_{p}^{*}}\cdots \int_{\mathbb{Z}_{p}^{*}} \bigl\vert s(t_{1}) \bigr\vert _{p} ^{-n_{1}/q}\cdots \bigl\vert s(t_{m}) \bigr\vert _{p}^{-n_{m}/q}\varphi (t_{1},\ldots,t _{m})\,dt_{1}\cdots{dt_{m}}< \infty. $$

### Remark 3.2

Noting that Theorem 3.3 is a more general result than the above two theorems, the operator $\mathcal{H}_{\varphi,m} ^{p,s}$ is reduced to the weighted Hardy type operator and its dual operator if $s(t)$ is equal to some suitable functions. The sufficiency of Theorem 3.3 can be obtained easily by the generalized Minkowski inequality and a *p*-adic change of variables. It is worth pointing out that we need to make some appropriate modifications for the necessity. Applying this condition $\vert s(t) \vert _{p}\geq \vert t \vert _{p}^{\beta}$, then the area of $s(t)$ is converted into the relevant area of *t*, so it is easy to get the desired results by adopting the same method as Theorem 3.1.

## Conclusions

In the present study, we introduced a class of *p*-adic Hardy type operator and considered the problem of finding a bound for the norm of it from *p*-adic Lebesgue product spaces with power weights to itself. Moreover, we characterized a sufficient and necessary condition which ensures that the weighted *p*-adic Hardy type operator is bounded on the *p*-adic Lebesgue product spaces. In addition, we also extended the results to the *p*-adic weighted Hardy-Cesàro operator.
